# A comparative analysis of unintegrated HIV-1 DNA measurement as a potential biomarker of the cellular reservoir in the blood of patients controlling and non-controlling viral replication

**DOI:** 10.1186/s12967-020-02368-y

**Published:** 2020-05-19

**Authors:** Chiara Orlandi, Benedetta Canovari, Federica Bozzano, Francesco Marras, Zeno Pasquini, Francesco Barchiesi, Andrea De Maria, Mauro Magnani, Anna Casabianca

**Affiliations:** 1 Department of Biomolecular Sciences, University of Urbino “Carlo Bo”, Urbino, Italy; 2 Malattie Infettive, Azienda Ospedaliera Ospedali Riuniti Marche Nord, Pesaro, Italy; 3 Ospedale Bambin Gesù, Rome, Italy; 4 Division of Infectious Diseases, Ospedale Policlinico S. Martino IRCCS, Genoa, Italy; 5 Dipartimento di Scienze Biomediche e Sanità Pubblica, Università Politecnica delle Marche, Ancona, Italy; 6 Department of Health Sciences, DISSAL, University of Genova, Genoa, Italy

**Keywords:** HIV-1, Total HIV DNA, Unintegrated HIV DNA, 2-LTR circles, Reservoir, qPCR, Blood cells, Viremic patients, Aviremic patients, HIC

## Abstract

**Background:**

The persistence of HIV-1 in reservoir cells is one of the major obstacles to eradicating the virus in infected individuals receiving combination antiretroviral therapy (ART). HIV-1 persists in infected cells as a stable integrated genome and more labile unintegrated DNA (uDNA), which includes linear, 1-LTR and 2-LTR circular DNA. 2-LTR circle DNA, although less abundant, is considered a surrogate marker of recent infection events and is currently used instead of the other unintegrated species as a diagnostic tool. This pilot study aimed to investigate how to best achieve the measurement of uDNA.

**Methods:**

A comparative analysis of two qPCR-based methods (U-assay and 2-LTR assay) was performed on the blood of 12 ART-naïve, 14 viremic and 29 aviremic On-ART patients and 20 untreated spontaneous controllers (HIC), sampled at a single time point.

**Results:**

The U-assay, which quantified all unintegrated DNA species, showed greater sensitivity than the 2-LTR assay (up to 75%, p < 0.0001), especially in viremic subjects, in whom other forms, in addition to 2-LTR circles, may also accumulate due to active viral replication. Indeed, in aviremic On-ART samples, the U-assay unexpectedly measured uDNA in a higher proportion of samples (76%, 22/29) than the 2-LTR assay (41%, 12/29), (p = 0.0164). A trend towards lower uDNA levels was observed in aviremic vs viremic On-ART patients, reaching significance when we combined aviremic On-ART and HIC (controllers) vs Off-ART and viremic On-ART subjects (non-controllers) (p = 0.0003), whereas 2-LTR circle levels remained constant (p ≥ 0.2174). These data were supported by the high correlation found between uDNA and total DNA (r = 0.69, p < 0.001).

**Conclusions:**

The great advantage of the U-assay is that, unlike the 2-LTR assay, it allows the accurate evaluation of the totality of uDNA that can still be measured even during successful ART when plasma viremia is below the cut-off of common clinical tests (< 50 copies/mL) and 2-LTR circles are more likely to be under the quantification limit. UDNA measurement in blood cells may be used as a biomarker to reveal a so far hidden or underestimated viral reservoir. The potential clinical relevance of uDNA quantification may lead to improvements in diagnostic methods to support clinical strategies.

## Background

Current antiretroviral therapy (ART) successfully suppresses viral replication below the detection limit of current clinical tests (< 50 HIV-1 RNA copies/mL) and reduces HIV-1 transmission, but fails to completely eliminate HIV-1. Indeed, persistent low-level HIV-1 can still be detected in plasma and cellular reservoirs even after years of ART. HIV-1 persists predominantly within circulating and tissue-resident long-lived memory CD4+ T cells that harbor HIV DNA [1, 2], while other less accessible reservoirs have been described [3–5], but represent a minor quantitative proportion of the total HIV-1 burden. CD4+ T cells harboring HIV DNA are an acknowledged source of viral rebound when treatment is discontinued, preventing complete virus eradication in patients treated with current therapies [6, 7].

The first event of the HIV-1 life cycle is the reverse transcription of viral RNA into a double-stranded linear DNA, which appears in the cytoplasm within hours after cell infection and is then transported into the nucleus. HIV DNA accumulates in infected cells as various species of DNA, as both integrated provirus and unintegrated genomes (uDNA). Integrated viral DNA is essential to a productive infection [8, 9] and persists for the life of the infected cell and its descendants, proving that integration is a major factor in HIV-1 persistence, even though it includes both replication-competent and defective genomes [10–12]. The uDNA exists in linear and circular DNA forms. The latter contain one long terminal repeated (LTR) copy (1-LTR) or two LTR copies (2-LTR) generated by ligation of both ends of the linear precursor by the host cell non-homologous end joining (NHEJ) pathway and other rearranged circular species [13–15]. Some of these forms derive from auto-integration events or error-prone reverse transcription reactions leading, for instance, to 2-LTR circles harboring imperfect palindromic junctions in contrast to 2-LTR circles originating from NHEJ [16]. The uDNA is considered a dead-end product of failed infections, but, recent evidence has shown that uDNA can be transcriptionally active, and production of early viral RNA and proteins has been observed in several cell types, including resting CD4 T cells [17–22]. Interestingly, recent data suggest that 2-LTR circle DNA may constitute a potential reserve supply of genomes for de novo integration [23].

In clinical practice there is a pressing need to identify the optimal biomarkers to measure the size and dynamics of the HIV DNA reservoir in the various stages of infection both in view of immunotherapy and eradication attempts and the care of patients. Several protocols for measuring persistent HIV-1 based on cell culture and qPCR assays have been developed [24]. One currently used qPCR-based approach quantifies total HIV DNA from the infected blood cells (both latently and productively infected cells) of patients, and although it is presently considered adequate for the assessment of HIV reservoir size in routine clinical practice [25, 26], it is unable to distinguish integrated from unintegrated forms of HIV DNA. Other qPCR-based assays have been developed to measure integrated HIV-1 DNA [27–33] and unintegrated DNA, by estimating episomal 2-LTR [30, 32, 34–39] as well as cell-associated unspliced RNA (usRNA) [40]. As for total HIV DNA, assays measuring intracellular HIV RNA [24, 41, 42] may overestimate the size of the viral reservoir because these assays are unable to distinguish between intact replication-competent and defective virus [11, 43, 44]. With regard to the impact of ART treatment on HIV DNA levels, there is conflicting evidence in the literature. There are some reports of total HIV DNA levels approaching those of integrated HIV DNA in well-controlled patients on ART [45], while other authors report an excess of unintegrated HIV DNA in treated and elite suppressor patients [46, 47]. Because of this uDNA excess, total HIV DNA does not always correlate with the level of integrated HIV DNA and thus may not always be the ideal clinical parameter to evaluate reservoir size in all patients.

Furthermore, uDNA is assessed by quantifying 2-LTR circle DNA despite the fact that it is present in lower levels than other unintegrated DNA species [29, 48, 49]. In fact, its relative scarcity might limit the ability of researchers to evaluate 2-LTR circle DNA, especially when the plasma viral load is very low. The 2-LTR species have been shown to be labile and are used as a surrogate marker of recent infection events [50–52] thus providing insight into reservoir dynamics. However, there is still some controversy around the use of 2-LTR species in this regard, with other reports showing that 2-LTR circles are stable in vitro and persist for a long time in infected cells [35, 38, 53].

Our group has developed two qPCR-based assays: the first (*TotUFsys* platform, called the U-assay in this paper) is able to simultaneously and directly measure total HIV DNA and the totality of uDNA in white blood cells (WBC) using a single set of primers targeting one of the most conserved HIV-1 genome regions, while the second (2-LTR assay) is able to specifically quantify 2-LTR circles using primers designed in the unique sequence junction formed upon end-to-end joining of the linear genome [30]. We previously showed that analyzing uDNA levels rather than just 2-LTR circle DNA seemed to be a more effective approach to reduce the percentage of undetected samples or samples near the low quantification limit from 53 to 29% [30]. However, to our knowledge, no study to date has directly measured uDNA in infected blood cells of HIV-1 patients with different levels of virological control. Labile unintegrated forms have recently been determined by calculating the difference in the number of copies between total and integrated HIV DNA [54].

In the present pilot study we compare the accuracy of the U-assay and the 2-LTR assay in detecting and quantifying the true excess of unintegrated species and the contribution of uDNA and 2-LTR circles to total HIV DNA in the blood samples of 75 HIV-1 patients controlling or non-controlling viral replication either spontaneously or after ART.

We showed that uDNA measurement improves the limit of detection of unintegrated DNA forms in infected cells even below the limit of detection of the 2-LTR method in aviremic patients and enhances the precision of the actual DNA reservoir detection.

## Materials and methods

### Study subjects

Seventy-five HIV-1 patients were recruited between 2009 and 2015 from clinical centers in Liguria (Ospedale Policlinico San Martino, Genoa; Ospedale Galliera, Genoa; Ospedale Sanremo, Sanremo), Piedmont (Ospedale Amedeo di Savoia, Turin) and the Marches (Azienda Ospedaliera Ospedali Riuniti Marche Nord, presidio San Salvatore, Pesaro), Italy. In this pilot study, whole blood samples were collected at a single time point from: (1) ART-naïve patients (Off-ART, n = 12), who were sampled during the acute or chronic phase of infection; (2) ART-treated patients with detectable plasma HIV-1 RNA above 50 copies/mL (viremic On-ART, n = 14); (3) ART-treated patients who had plasma HIV-1 RNA below 50 copies/mL (aviremic On-ART, n = 29); (4) elite controllers (EC) and long-term non-progressors (LTNP) pooled and analyzed as HIV-infected controllers (HIC, n = 20; Total HIV DNA, uDNA, 2-LTR circles of EC vs LTNP: p ≥ 0.4972, Additional file 1: Table S1). EC subjects had positive HIV-1 serology, were ART-naïve, and had CD4+ counts ≥ 450 cells/μL for ≥ 7 years of follow-up with no clinical evidence of disease progression. LTNP met the same definitions as ECs, except for the fact that HIV-1 RNA was detectable during the years of observation and < 2000 copies/mL.

Baseline characteristics and clinical parameters (time since diagnosis, duration of ART, CD4+ T cell count and plasma viremia at sampling) of the patient cohorts are summarized in Table 1 and Additional file 2: Table S2. The HIC patients were previously described and analyzed by Marras et al. [55], now HIV DNA levels were quantified in whole blood samples. Measurements (plasma HIV-1 RNA, CD4+ count and HIV DNA levels) from 8 of the Off-ART subjects, 7 of the viremic On-ART subjects and 15 of the aviremic On-ART subjects were previously reported in Casabianca et al. [30]. All measurements were used for comparative analyses of the groups. The percentage of uDNA among total HIV DNA, the percentage of 2-LTR circles among total HIV DNA and the percentage of 2-LTR circles among the uDNA were assessed for the first time in the present study. When appropriate, we combined the results from Off-ART and viremic On-ART subjects and those from aviremic On-ART and HIC subjects, and referred to them as “non-controllers” and “controllers”.

**Table 1:** Clinical characteristics of patient cohorts

Characteristics	Overall (n = 75)	Off-ART (n = 12)	On-ART (n = 43)	Among On-ART		HIC (n = 20)	p value
				Viremic (HIV-1 RNA > 50 cp/mL) (n = 14)	Aviremic (HIV-1 RNA < 50 cp/mL) (n = 29)		
Time since diagnosis (years) NA		0.5 [0–3]	2 [1–9] 21	1 [0–1] 7	4 [1–15] 14	> 7	0.0043^a^
Time since ART initiation (months) NA		n/a	24 [1–75] 21	1 [0.5–1] 7	36 [5–156] 14	n/a	0.0030^a^
CD4+ count (cells/µL)	460 [295–751]	373 [318–491]	325 [272–535]	285 [185–416]	360 [293–679]	761 [603–1047]	< 0.0001
HIV-1 RNA (copies/mL of plasma)	50 [26–10^3^]	10^5^ [10^5^–7.75*10^5^]	50 [18–110]	10^3^ [108–10^3^]	50 [4–50]	172 [20–1214]	< 0.0001
Total HIV DNA							
(copies/µg DNA)	20 [8–38]	33 [26–43]	21 [16–40]	22 [13–56]	21 [16–40]	8 [3–13]	< 0.0001
(copies/10^4^ CD4+)	17 [7–35]	27 [19–36]	26 [11–48]	16 [9–55]	27[13–49]	5 [2–8]	< 0.0001
uDNA							
(copies/µg DNA)	4 [1–11]	11 [7–14]	7 [3–13]	11 [5–17]	5 [2–11]	1 [1–2]	< 0.0001
(copies/10^4^ CD4+)	5 [1–10]	8 [5–11]	6 [2–13]	9 [5–16]	5 [1–12]	1 [1]	< 0.0001
2-LTR circles							
(copies/µg DNA)	1 [1–2]	1 [1–5]	1 [1–3]	1 [1–2]	1 [1–4]	1 [1–2]	0.7171
(copies/10^4^ CD4+)	1 [1–2]	1 [1–5]	1 [1–3]	1 [1–2]	1 [1–5]	1 [1]	0.2174
Percentage of uDNA among total HIV DNA	26 [5–42]	37 [28–41]	30 [13–46]	45 [22–80]	22 [5–38]	0 [0–23]	0.0013
Mean ± SEM	28 ± 3	33 ± 3	32 ± 4	49 ± 9	24 ± 4	15 ± 5	
Percentage of 2-LTR circles among total HIV DNA	0 [0–18]	0 [0–17]	0 [0–18]	0 [0–16]	0 [0–18]	0 [0–18]	0.8684
Mean ± SEM	10 ± 2	6 ± 3	11 ± 3	14 ± 8	9 ± 2	11 ± 4	
Percentage of 2-LTR circles among uDNA	0 [0–82]	0 [0–45]	0 [0–76]	0 [0–43]	62 [0–92]	84 [68–92]	0.0024
Mean ± SEM	38 ± 6	17 ± 9	36 ± 7	17 ± 8	47 ± 10	75 ± 10	
Percentage of iDNA among total HIV DNA							
Using U-assay	75 [59–96]	63 [59–72]	70 [54–87]	56 [21–78]	78 [63–96]	100 [77–100]	0.0013
Mean ± SEM	72 ± 3	67 ± 3	68 ± 4	51 ± 9	76 ± 4	85 ± 5	
Using 2-LTR assay	100 [82–100]	100 [83–10]	100 [82–100]	100 [84–100]	100 [82–100]	100 [82–100]	0.8684
Mean ± SEM	90 ± 2	94 ± 3	89 ± 3	86 ± 8	91 ± 2	90 ± 4	
p value^b^	< 0.0001	0.0025	< 0.0001	0.0005	0.0004	0.0138	

*NA* data not available, *n/a* not applicable

The most common ART regimen was a combination of 2NRTIs + PI (41%); 10 subjects were on an INI-based regimen (CD4+ count, HIV-1 RNA, Total HIV DNA, uDNA, 2-LTR circles vs other regimens: p ≥ 0.1323)

Data are medians [25^th^–75^th^ percentile], unless otherwise indicated. The Kruskal–Wallis test or ^a^ Mann–Whitney test between viremic and aviremic On-ART patients

The contribution of integrated HIV DNA (iDNA) to total HIV DNA was calculated by the difference in the percentage between total (100%) and uDNA or 2-LTR circles among total HIV DNA, ^b^ Wilcoxon signed rank test

### Measurements of HIV-1 DNA

The detailed procedure for HIV DNA level measurement has been previously described [30, 56]. Briefly, we reported the workflow of experiments and any changes or improvements in the procedure. Cellular DNA was isolated and purified from WBC of frozen blood samples (1 mL). After incubation of the WBC pellet for 45 min at 37 °C in a lysis buffer, the DNA was purified by phenol extraction, ethanol precipitation and RNase treatment, ensuring adequate amounts of concentrated (mean DNA recovery (SD): 19.6 (7.4) µg/mL) high purity DNA (absorbance ratios A260/A280: ≥ 1.8, NanoVue Plus spectrophotometer, GE Healthcare) for all PCR reactions. Each sample was analyzed with two different SYBR Green qPCR based methods: *i)* we used the *TotUFsys* platform (U-assay in the present study) for total HIV DNA and unintegrated HIV DNA (the ensemble of extrachromosomal viral cDNAs, including both linear cDNA and all the closed circular 1-LTR and 2-LTR and other rearranged forms); *ii)* we used the 2-LTR assay for unintegrated 2-LTR circular forms [30]. The totality of uDNA was obtained from cellular DNA by an optimized chromatographic procedure able to separate the high molecular weight DNA from the low molecular weight DNA (consisting of uDNA present in the eluate fraction). Appropriate control experiments on the chromatographic separation have demonstrated the feasibility of the procedure [30, 56].

**Fig. 1:**
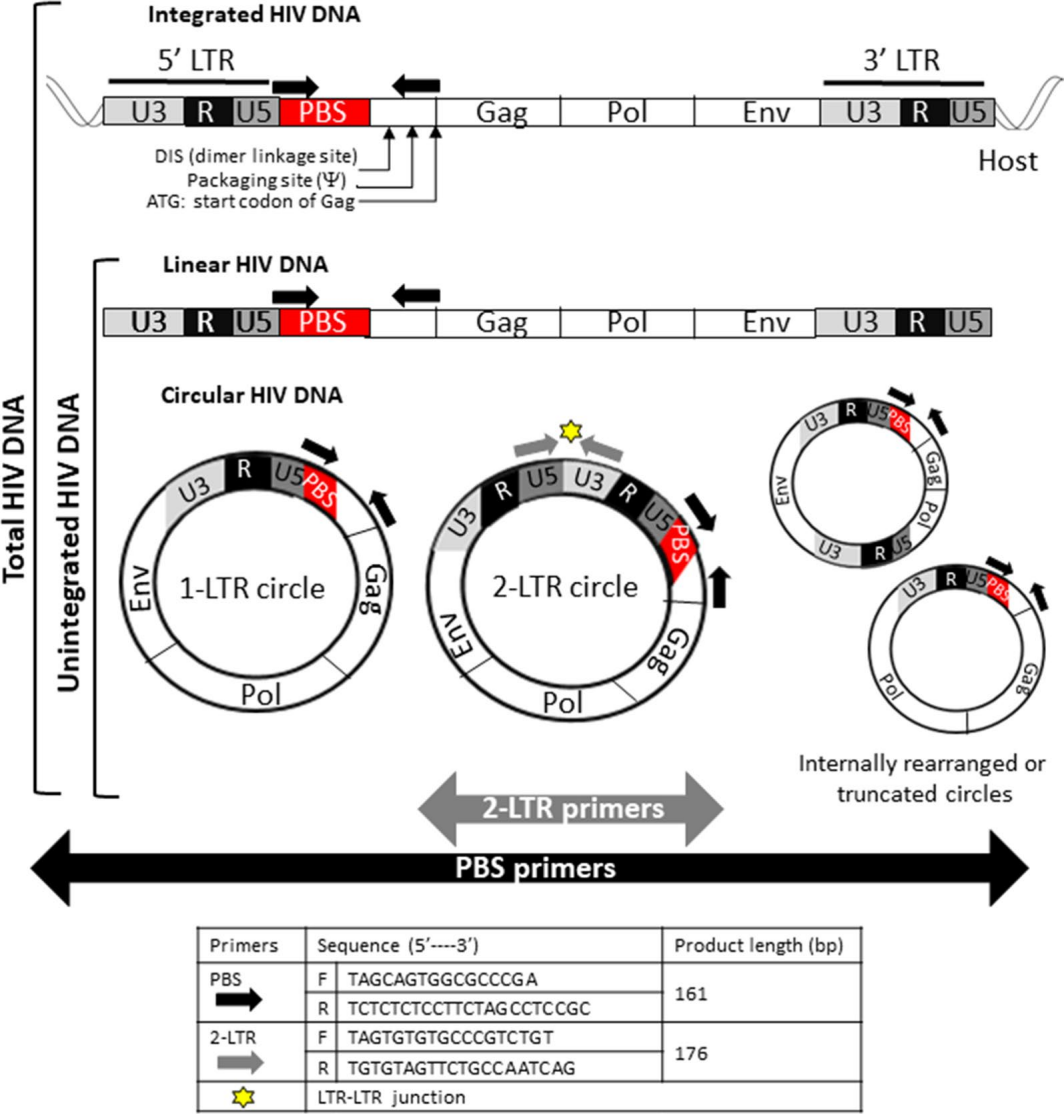
Real-time PCR strategy for the quantification of various HIV-1 DNA species. Approximate locations of forward and reverse primers used for the amplification of both total HIV-1 DNA (integrated and unintegrated species) and unintegrated forms (PBS primers indicated in black) and for the selective amplification of 2-LTR circles (2-LTR primers indicated in grey) are shown. The table shows the legend and sequences of primers used. In the U-assay, the QIAamp plasmid Mini Kit (Qiagen) was used to separate the low molecular weight DNA (LMW, consisting of unintegrated HIV DNA species) from the high molecular weight DNA (HMW, containing the integrated proviral HIV DNA). In the 2-LTR assay, the elongation time of 35 s, is not compatible with a reliable amplification of non-specific products. The HIV-1 DNA genome is not drawn to scale

Total and unintegrated HIV DNA were simultaneously analyzed by qPCR in a single run using a single set of specific primers selected in the 5′ LTR-Gag region of the HIV-1 genome, including the highly conserved primer-binding site (PBS) and able to detect all HIV-1 subtypes in the M group. The qPCR measurements of 2-LTR circles were performed using primers targeting the dual-repeat cassette within the circular forms (Fig. 1). For both assays, the quantification range was 5-log, the quantification limit (QL) was 2 copies/μg of DNA, while the detection limit was 1 copy/μg. Reproducibility, analyzed as intra- and inter-assay variability, was 20%, confirming the accuracy of the technical setting in the full dynamic range of quantification.

PCR reactions were carried out in a 7500 real-time PCR system (Applied Biosystems, Thermo Fisher Scientific Inc.), using the Hot-Rescue Real-Time PCR Kit Sybr Green (Diatheva srl). Each sample (cellular DNA or eluate fraction containing uDNA) was analyzed in six replicates, consisting of three wells containing 0.5 µg of DNA and three wells containing 1.0 µg of DNA (4.5 µg, to ensure the detection of the target even in the low copy number, i.e. near the QL) for total HIV DNA, uDNA and 2-LTR circle measurements. For samples with HIV DNA datum quantified near or detected below the QL, two additional 1 µg replicates were tested for a total of 6.5 µg of DNA (~ 10^6^ WBC). In the case of negative amplification, a PCR spike test was performed by adding two or ten copies of plasmid standard to the samples to exclude the presence of inhibitors. Total, unintegrated and 2-LTR circle HIV DNA copy numbers were estimated by interpolation of the experimentally determined threshold cycle (Ct) based on the plasmid reference standard curves generated using half-log serial dilutions from 10^3^ to 2 copies to obtain a more precise standard in the low range of quantification. The pPBS plasmid contained a 161 bp-PBS fragment derived from HIV-1 PNL4-3 vector cloned into the pGEM-T vector and the p2LTR plasmid was obtained by cloning a 176 bp fragment of the LTR–LTR junction within the 2-LTR circular form in a pGEM-T vector. Standard curves were created automatically and accepted when the PCR efficiency was 97–100% and the minimum value of the correlation coefficient (R^2^) was 0.98. The percentage of amplification efficiency was estimated based on the slope value from the linear regression (log of copy number vs Ct values) as [10^(− 1/slope)^− 1] × 100. The similar PCR efficiency of the two standards in their linear range of quantification allowed us to perform accurate data comparisons (∆slope ≤ 0.1, [30, 57]).

Total, unintegrated, and 2-LTR circle HIV DNA copy numbers were determined by adding up the copy number from the 0.5 and 1.0 µg replicates tested and normalized to 1 µg of DNA and then expressed as copies/10^4^ CD4+ T cells. This approach was based on the assumption that 1 µg of genomic DNA corresponds to 142857 cells [58] and that HIV DNA is present principally in CD4+ T lymphocytes [6]. Hence, the following formula was used: [(copies/µg DNA)/(CD4+ T cell count (/µL)/WBC count (/µL) × 142857 WBC)] × 10^4^. For samples with an HIV DNA target below the QL (i.e. < 2 copies/µg), an imputed value corresponding to ½QL (1 copy) was used for subsequent normalization and statistical analyses.

### 2-LTR circle junction sequencing

PCR products (176 bp) from blood DNA samples of six aviremic patients were separated on 2% agarose gel and purified using MinElute Gel Extraction Kit (Qiagen). DNA sequencing was performed using the BigDye Terminator v. 1.1 Cycle Sequencing Kit on an ABI PRISM 310 Genetic Analyzer (Applied Biosystems).

### Quantification of plasma HIV-1 RNA and CD4+ T cell count

Plasma HIV-1 RNA levels were quantified using commercial kits according to the manufacturer’s instructions (artus HI Virus-1 QS-RGQ Kit Qiagen, or Nuclisens EasyQ HIV-1 2.0 bioMérieux SA). CD4+ lymphocyte counts were determined using flow cytometry analysis.

### Statistical analysis

The data are presented as the median and interquartile range (IQR, 25th to 75th percentile), unless indicated otherwise. All comparisons between the different groups were made using the appropriate nonparametric tests (the Mann–Whitney U test for two-group unpaired data or the Kruskal–Wallis test for comparisons of more than two groups followed by Dunn’s Multiple Comparison test). The Wilcoxon signed rank test was used for matched samples. Correlations were investigated using Spearman’s nonparametric correlation test. Fisher’s exact test was used to compare the detectability of unintegrated HIV DNA in patient samples obtained using the two methods. A p value below 0.05 (two-sided) was considered statistically significant.

## Results

### Sensitivity differences between the two versions of the assay for uDNA measurement

**Fig. 2:**
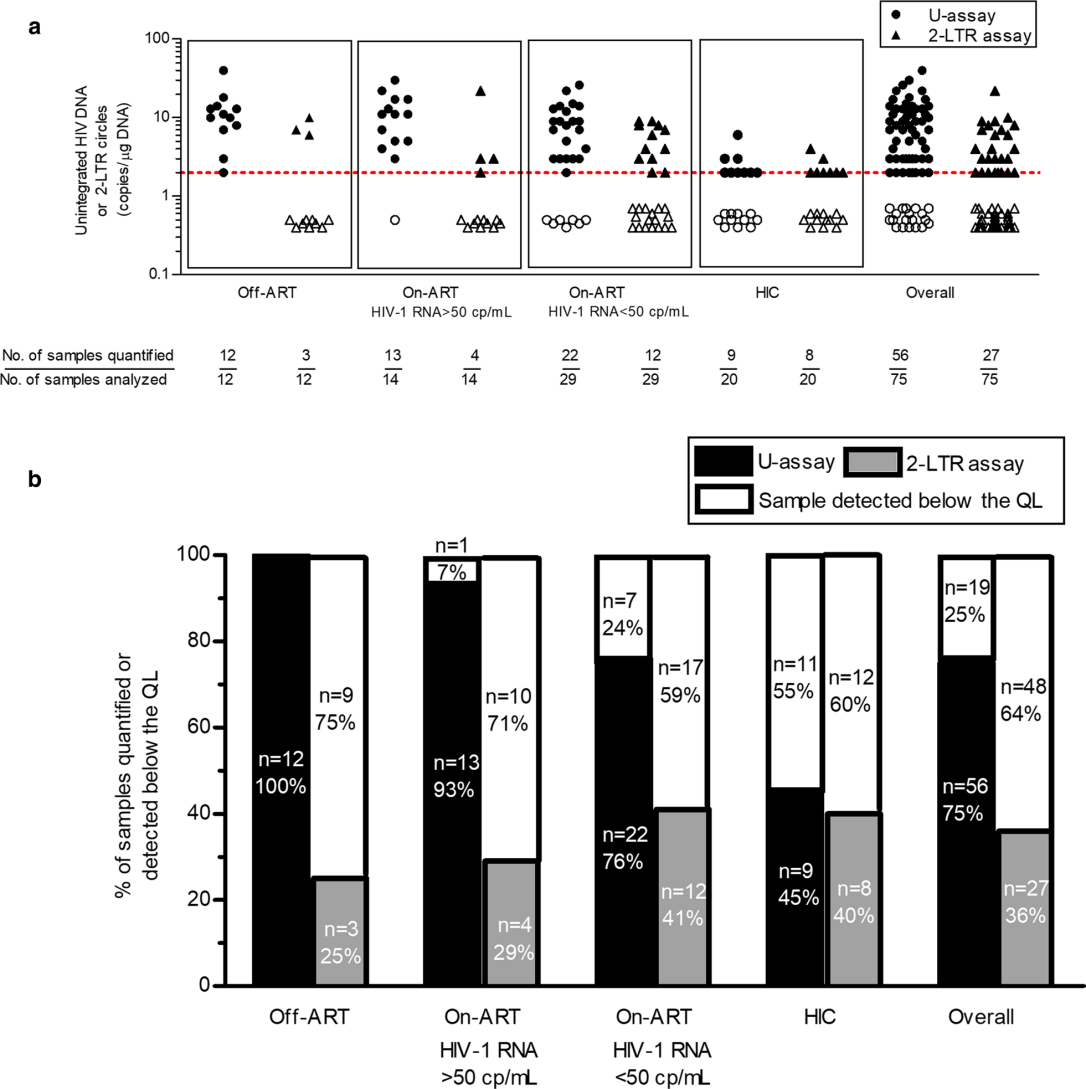
Sensitivity differences between the U-assay and 2-LTR assay for unintegrated HIV DNA detection. **a** Quantification of unintegrated HIV DNA and 2-LTR circles in blood samples of Off-ART patients (n = 12), viremic (n = 14) and aviremic (n = 29) On-ART patients, and untreated infected controller patients (HIC, n = 20). The dotted line indicates the limit of quantification (QL, 2 copies). Detectable values below the QL are depicted by empty symbols. **b** Proportion of samples from the four groups of HIV-1 patients, with quantifiable (colored bars) or detectable (white bars) levels of unintegrated HIV DNA (black) and 2-LTR circles (gray). The number of samples from each group quantified or detected below the QL by each assay is indicated. The frequency with which the levels of unintegrated viral DNA were quantified was higher using the U-assay than the 2-LTR assay (overall p < 0.0001, Fisher’s exact test)

To examine the sensitivity of the U-assay and 2-LTR assay for uDNA quantification (copy number/µg of cellular DNA), we used blood samples from Off-ART, viremic On-ART, aviremic On-ART as well as HIC patients (Fig. 2, Additional file 2: Table S2). Firstly, the U-assay yielded a substantial increase in the number of samples that could actually be quantified, showing an overall higher quantification frequency of 39% (29/75, p < 0.0001). In particular, using the U-assay we were able to quantify unintegrated forms in almost all of the samples from Off-ART (100%, 12/12) and On-ART viremic (93%, 13/14) subjects. Unexpectedly, we were able to measure uDNA in a high proportion of samples from On-ART aviremic patients (76%, 22/29). On the other hand, we found a lower rate of quantified samples using the 2-LTR assay: 25% (3/12), 29% (4/14) and 41% (12/29) for Off-ART, On-ART viremic and On-ART aviremic patients, respectively. In HIC patients, the two assays yielded similar results (45%, 9/20 and 40%, 8/20 for the U-assay and 2-LTR assay, respectively). Thus, a net gain in the proportion of samples that could be readily quantified was obtained using the U-assay, particularly in viremic subjects both Off- and On-ART (75% 9/12, p = 0.0003; 64% 9/14, p = 0.0013; 35% 10/29, p = 0.0164; for the Off-ART, viremic On-ART and aviremic On-ART groups, respectively) with a smaller net gain in HIC subjects (5% 1/20, p = 1). Secondly, an HIV DNA datum quantified near or below the QL required further investigation (see Materials and methods section); hence, using the 2-LTR assay 77% of the samples (58/75) had to undergo a second PCR run, with a consequent increase in reagent costs and the time required to perform the analyses. On the hand, using the U-assay, a lower percentage of samples (30/75, 30%) required two additional 1 µg replicates (2nd PCR) (p < 0.0001; Fisher’s exact Test, 2-tailed). It should be noted that the U-assay requires a chromatographic DNA isolation procedure, which takes about 40 min, and analysis of the level of contamination with chromosomal DNA, less time, in any case, than is spent for the second PCR (~ 1 h for the set up and ~ 2 h for the PCR run) required for most of samples tested by the 2-LTR assay.

### DNA sequence variability of the 2-LTR circle junction

DNA sequence analyses have revealed that in 40–50% of in vitro infected cells and in up to 88% of patient PBMCs, the 2-LTR circle junctions are mutated with deletions, insertions, single nucleotide mutations, etc. lacking the normal unprocessed ends for canonical 2-LTR circle formation (GTAC) [59, 60]. All the sequence data from our six aviremic On-ART patients displayed sequence heterogeneity across the circle junction. In Additional file 3: Figure S1, some examples of sequence variability of the LTR–LTR junction are shown. In panel A, the circle junction region lacks GT dinucleotide, while in panel B and C, we can observe high variability in the DNA sequence.

### Quantification of total and unintegrated HIV DNA in samples from HIV-infected subjects

**Fig. 3:**
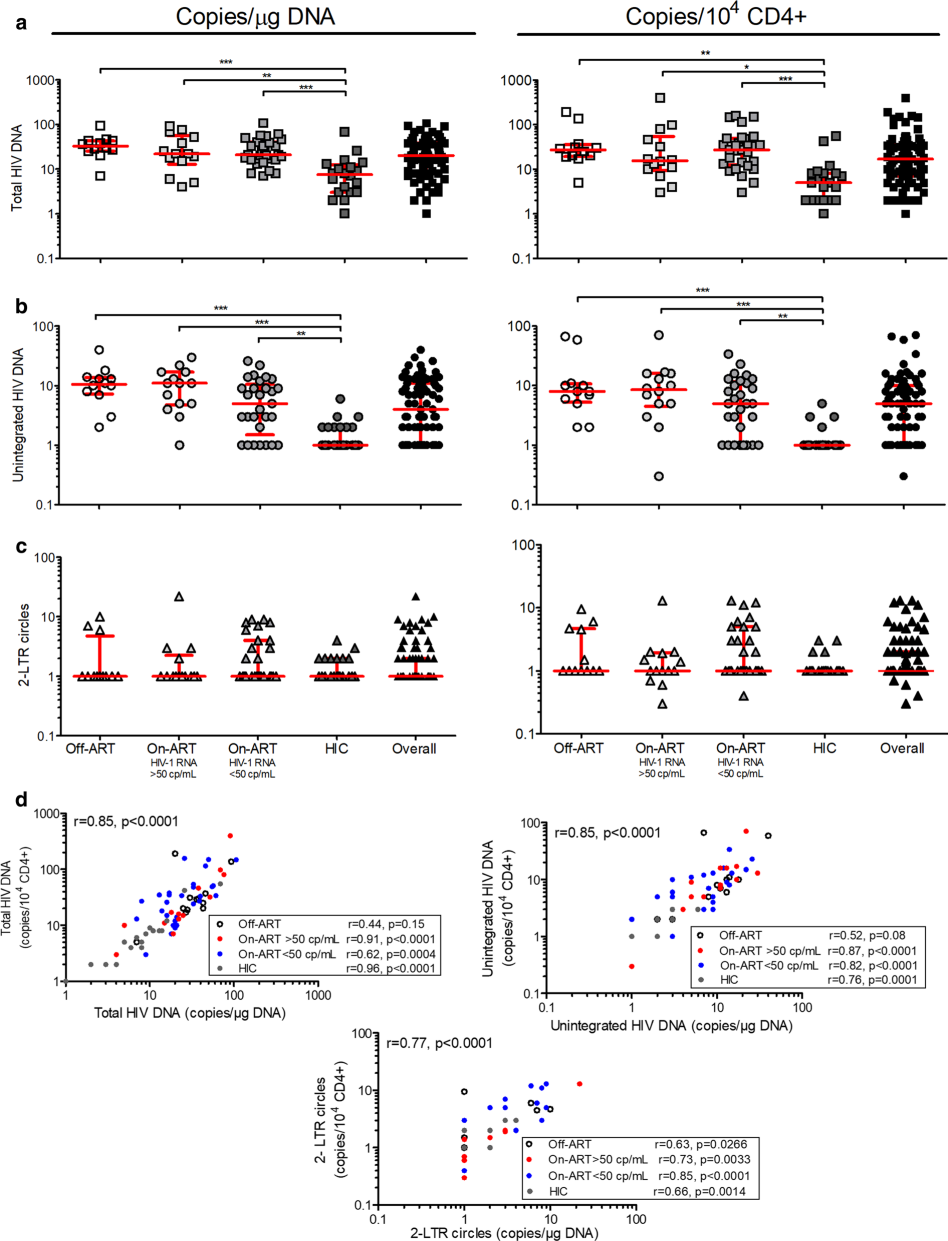
Quantification of total and unintegrated HIV DNA in blood samples from HIV-1 infected patients. Levels of **a** total HIV DNA, **b** unintegrated HIV DNA and **c** 2-LTR circles were measured in Off-ART patients (n = 12), viremic (n = 14) and aviremic (n = 29) On-ART patients, and untreated infected controller patients (HIC, n = 20). Each DNA sample was tested using three 0.5-μg replicates and three (or up to five) 1.0-μg replicates for a total of up to 6.5 µg (~ 10^6^ WBC). The number of copies was reported per µg of cellular DNA (left) and per 10^4^ CD4+ (right) converting the value relative to 1 µg of cellular DNA for the CD4+ count and the proportion of CD4+ T cells in each subject. Lines represent the median and 25th to 75th percentiles. The Kruskal–Wallis test and Dunn’s multiple comparison test were used for comparisons, only p < 0.05 are reported; *p < 0.05; **p < 0.01; ***p < 0.001. **d** Correlations between the levels of total HIV DNA, unintegrated HIV DNA and 2-LTR circles expressed as copy number per µg of cellular DNA or per 10^4^ CD4+ T cells in the four different groups of HIV-1 patients. Correlations were assessed by Spearman’s rank-correlation coefficient. The correlation coefficients and p-values are shown in each plot

HIV DNA quantification was performed on blood cells, bypassing PBMC separation. We were able to normalize the data in relation to a constant number of CD4+ T cells, assuming that most (> 98%) of the HIV DNA is detected in CD4+ T cells [6] and also taking into account the variation in CD4+ T cell count during HIV infection and treatment. Indeed, HIV DNA levels expressed as copies/10^4^ CD4+ correlated strongly with those expressed as copies/µg of DNA for the 75 patients (r ≥ 0.77, p < 0.0001), and in each subgroup (p ≤ 0.003), with the exception of the Off-ART patients, which, given the small sample size of this group, failed to reach significance (p = 0.15) (Fig. 3d). Total HIV DNA was measurable in all 75 samples that were tested, with the exception of one HIC sample in which the amount was below the QL. Total HIV DNA levels ranged from < 2 to 107 copies per µg of DNA (corresponding to ~ 150000 WBC) and from 1 to 396 copies per 10^4^ CD4+. Unintegrated HIV DNA levels ranged from < 2 to 40 copies per µg and from 0.3 to 71 copies/10^4^ CD4+, with 48 (64%) samples quantified above the QL; 2-LTR values ranged from < 2 to 22 copies per µg and from 0.3 to 13 copies/10^4^ CD4+), with only 18 (24%) samples quantified above the QL (Fig. 3a–c, Additional file 2: Table S2). Compared to the Off- and On-ART groups, the HIC patients showed significantly lower levels of both total (3-5 fold lower, p < 0.0001) and uDNA (7–11 fold lower, p < 0.0001), while 2-LTR circles did not vary significantly among the groups (p ≥ 0.2174) (Fig. 3a–c, Table 1).

Analyses of pooled Off-ART and viremic On-ART patients (non-controllers) versus aviremic On-ART and HIC patients (controllers) showed no difference in total HIV DNA levels (median [IQR] 20 [13–39] vs 12 [5–35] copies/10^4^ CD4+, p = 0.0982), and significantly higher levels of uDNA in non-controllers than in controllers (8 [5–14] vs 2 [1–7] copies/10^4^ CD4+, p = 0.0003). No difference was found in 2-LTR amounts (p = 0.8095).

### Composition of HIV DNA in blood samples from HIV-1 infected patients

**Fig. 4:**
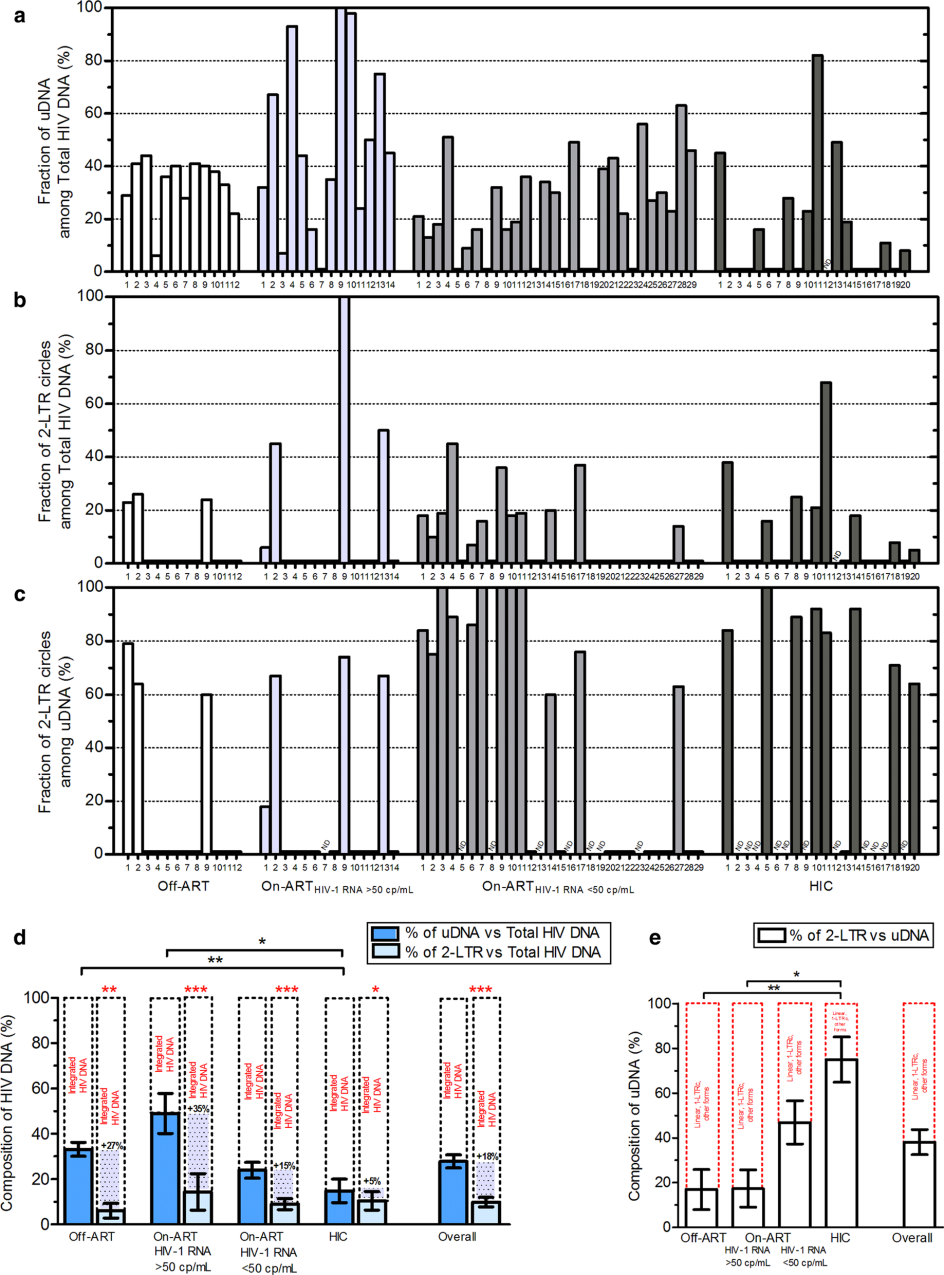
Composition of HIV DNA in blood samples from HIV-1 infected patients. Percentages of **a** uDNA among total HIV DNA, **b** 2-LTR circles among total HIV DNA and **c** 2-LTR circles among the totality of uDNA forms in Off-ART patients (n = 12), viremic (n = 14) and aviremic (n = 29) On-ART patients, and untreated infected controller patients (HIC, n = 20). Each bar within a group represents the datum from a single sample. The percentages refer to data expressed as HIV DNA copies per µg of DNA. The percentages of uDNA or 2-LTR circles in total HIV DNA and of 2-LTR circles in uDNA were reduced to 0% when uDNA or 2-LTR circle copy numbers were < QL of the assays. When both uDNA and 2-LTR circle copy numbers were < QL, the fractions are reported as non-determinable (ND). **d** The percentages from the above a-b panels are presented as mean ± SEM. Subtracting uDNA and 2-LTR circles from total HIV DNA, the remaining fraction is constituted by integrated HIV DNA (black dotted line). **e** The percentages from the above c panel are presented as mean ± SEM. Subtracting the 2-LTR circle DNA fraction from uDNA, the remaining fraction is constituted by the sum of 1-LTR circles, 2-LTR circles with anomalous junction sequence, rearranged circles and linear HIV DNA (red dotted line). The Kruskal–Wallis test and Dunn’s multiple comparison test were used for comparisons among groups of patients (black asterisks), the Wilcoxon signed rank test was used for matched samples (red asterisks), only p < 0.05 are reported; *p < 0.05; **p < 0.01; ***p < 0.001

Due to the high variability of the HIV DNA levels in our samples, the fractions of the unintegrated and 2-LTR HIV DNA within the total HIV DNA and of the 2-LTR circles within unintegrated DNA are shown for each subject (Fig. 4a–c). The percentage of uDNA among total HIV DNA varied from 0 to 100% within the samples (median [IQR]: 26% [5–42]). It was higher in non-controller patients, regardless of the treatment (p = 0.0013), and in aviremic On-ART patients, the percentage of uDNA was half of that found in viremic On-ART patients (22% [5–38] vs 45% [22–80]), (Fig. 4d, Table 1). Note that while the percentage of 2-LTR circles among total HIV DNA showed no difference among groups (range: 0-100%, median [IQR]: 0% [0-18]; p = 0.8684) (Fig. 4d, Table 1), the fraction of 2-LTR circles among uDNA was less than one-fifth in Off-ART and viremic On-ART patients and constituted a higher percentage of the uDNA in aviremic On-ART and HIC subjects (median [IQR]: 62% [0–92] and 84% [68–92], respectively; p = 0.0024), (Fig. 4e, Table 1).

Since the PCR efficiencies were high and similar to one another, by subtracting the uDNA or 2-LTR circle DNA fraction from total HIV DNA and 2-LTR circles from all unintegrated forms, we were able to provide the percentage of the integrated HIV DNA (iDNA) and the percentage of the sum of 1-LTR circles, 2-LTR circles with anomalous junction sequences, rearranged circles and linear HIV DNA, respectively. The latter percentage had a mean value of 83% in Off-ART and viremic On-ART patients (median [IQR]: 100% [55–100] and 100% [58–100]) and 53% and 25% in aviremic On-ART and HIC patients, respectively (median [IQR]: 39% [8–100] and 16% [8–33]), (Fig. 4e). The median fraction of uDNA within the total HIV DNA was significantly different from that of 2-LTR circle DNA within the total HIV DNA, both overall (p < 0.0001) and in each group of patient samples (p ≤ 0.0138). This observation revealed that, using the 2-LTR assay, the integrated fraction was overestimated by 27% to 35% in non-controllers and by 5% to 15% in controllers (p ≤ 0.0138), (Fig. 4d, Table 1).

Analyses of non-controllers versus controllers showed a higher percentage of uDNA among total HIV DNA in non-controllers (median [IQR]: 39% [27–46] vs 17% [0–34]; p = 0.0004) and a higher percentage of 2-LTR circles among the totality of unintegrated species in controllers (75% [0–92] vs 0% [0-39]; p = 0.0006), with no difference in the percentage of 2-LTR circles among the total HIV DNA (0% [0–39] vs 0% [0–18]; (p = 0.7170).

### Correlations between HIV DNA forms and plasma HIV-1 RNA and CD4+ T cell count

**Fig. 5:**
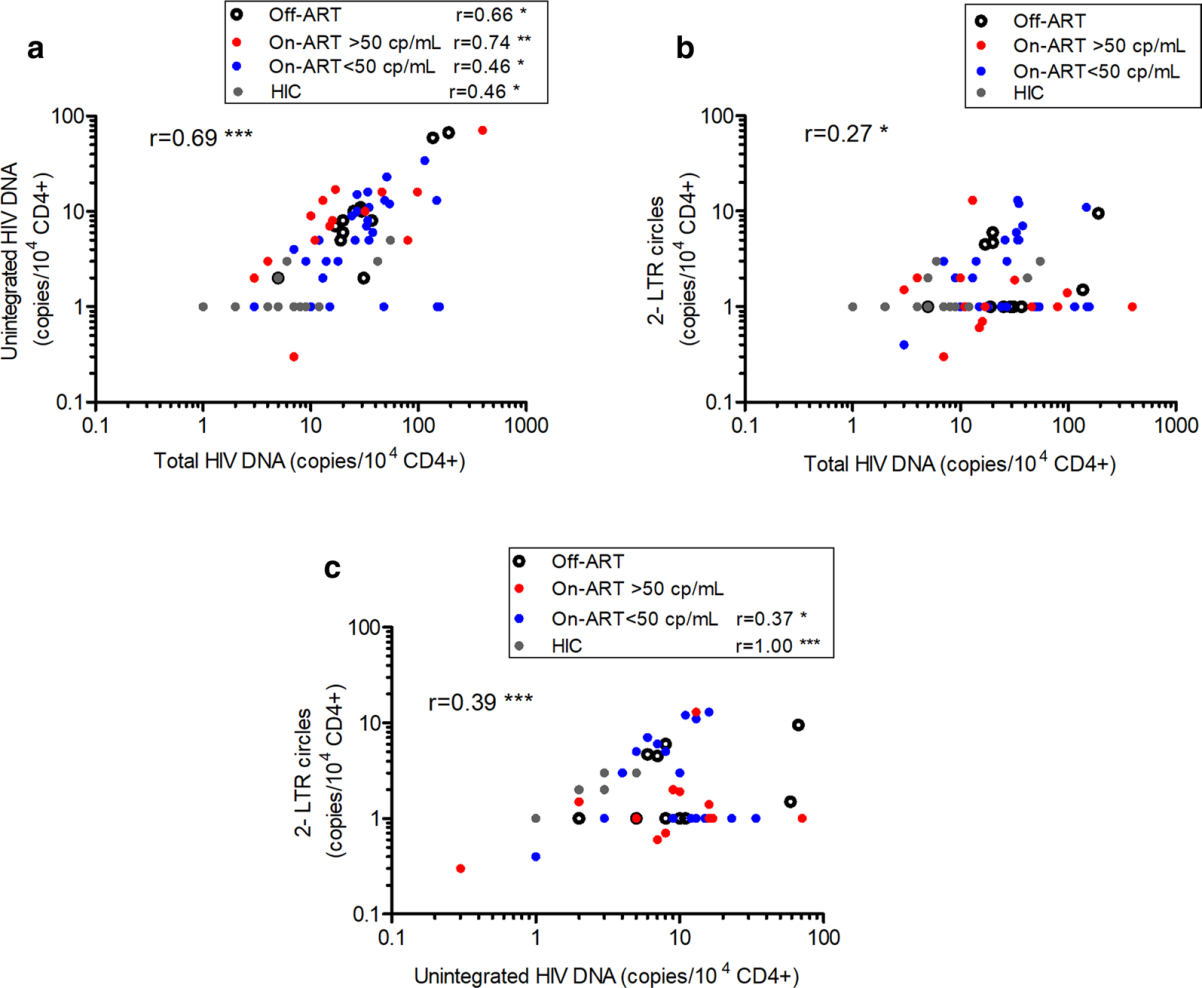
Correlations between the levels of the different forms of HIV DNA in blood samples from HIV-1 infected patients. Samples were obtained from Off-ART patients (n = 12), viremic (n = 14) and aviremic (n = 29) On-ART patients, and untreated infected controller patients (HIC, n = 20). Correlations were assessed by Spearman’s rank-correlation coefficient. The correlation coefficients and p-values are shown in each plot; *p < 0.05; **p < 0.01; ***p < 0.001

Associations between the uDNA, 2-LTR circle and total HIV DNA levels were investigated examining all the samples from the four different groups of patients and from each group separately (Fig. 5). Analyzing the whole sample population, we found a high positive correlation between uDNA and total HIV DNA levels (r = 0.69, p < 0.001), whereas 2-LTR circle DNA were more correlated with uDNA (r = 0.39, p < 0.001) than with total HIV DNA (r = 0.27, p = 0.018). High correlations between total and uDNA levels were observed for each subgroup, and viremic On-ART subjects showed the strongest correlation (r = 0.74, p = 0.002). A significant correlation between total HIV DNA and 2-LTR circle levels was not detected in any of the subgroup analyses, while 2-LTR circles were correlated with uDNA in aviremic On-ART patients (r = 0.37, p = 0.05) and more strongly correlated in HIC patients (r = 1.0, p < 0.001). The correlations between inferred iDNA levels, obtained from total HIV DNA copies minus uDNA copies, are shown in Additional file 4: Table S3.

No correlations between plasma HIV-1 RNA and HIV DNA levels were found (r ≤ 0.2, p ≤ 0.086), except in the Off-ART group, in which HIV-1 RNA positively correlated with both total (r = 0.65, p = 0.023) and unintegrated (r = 0.75, p = 0.005) HIV DNA, but not with 2-LTR circles (r = 0.34, p = 0.28), (Additional file 4: Table S4).

The associations with viremia found in the Off-ART group could be due to the fact that all these samples had quantifiable HIV-1 RNA, while overall, only 37 out 75 samples (49%) had > 50 copies/mL and the remaining 38 (51%) were derived from spontaneously or therapeutically well-suppressed patients. For 15 (39.5%) of these suppressed patients, the plasma viremia was simply recorded as < 50 copies/mL, while for 23 (60.5%), the residual viremia below 50 copies was specified since our blood samples were collected at several clinical centers that employed different commercial kits to quantify plasma HIV-1 RNA levels.

Overall, the total and uDNA levels were more strongly correlated (r = -0.68 and -0.59, p < 0.001) than the 2-LTR circles (r = -0.28, p = 0.016) with the CD4+ T cell count. In all the different subgroups, only the total HIV DNA levels, and not the unintegrated HIV DNA levels (p ≥ 0.07) or 2-LTR circles (p ≥ 0.06), were correlated with the CD4+ count (from r = -0.46 to r = − 0.68, p < 0.05), (Additional file 4: Table S5).

## Discussion

In this study, we have thoroughly analyzed two SYBR Green qPCR based assays (the U-assay and 2-LTR assay), assessing their ability to accurately measure the true excess of unintegrated HIV-DNA in infected blood cells. Given the similar high amplification efficiency and sensitivity of both assays, measurements of total and unintegrated HIV DNA and 2-LTR circle DNA can be accurately compared. We showed that in different patient groups (Off-ART, viremic and aviremic On-ART and HIC) the U-assay has greater sensitivity (about 40% and up to 75%) than the 2-LTR assay. The greater sensitivity of the U-assay was particularly evident in non-controllers, in whom, due to active cycles of replication, even linear DNA, 1-LTR and internally arranged circular forms are produced and go undetected by the 2-LTR assay. Moreover, the sequence analyses revealed heterogeneity across the 2-LTR circle junction in HIV-1 subjects [59, 60], which was confirmed in all our assayed aviremic On-ART patients. Although we cannot exclude the possibility that our 2-LTR SYBR Green I-based assay underestimated the level of 2-LTR anomalous DNA species due to poor annealing of primers and low reaction efficiency, the detection of mutated 2-LTR circle junctions is precluded by a qPCR that uses junctional probes and primers since this methodology is more sensitive to the presence of nucleotide mismatches in the target sequence [32, 34–39]. On the contrary, the U-assay, which uses primers hybridizing in a region conserved across clades of HIV-1 [61, 62] far from the 2-LTR junction sequence, can amplify the majority of unintegrated species, including the 2-LTR circles with anomalous junction sequences. Of note, using the U-assay, unintegrated HIV DNA could still be readily quantified in a higher percentage of blood samples (76%) than could be quantified using the 2-LTR assay (41%) in successfully treated patients.

Analyses of HIV DNA levels in the four cohorts of untreated and treated HIV-infected individuals showed that the amounts of total and unintegrated HIV DNA remain significantly higher, despite therapy, in On-ART patients than in HIC patients. Interestingly, a trend towards lower uDNA levels was observed in aviremic patients compared to viremic patients On-ART, whereas 2-LTR circle levels remained constant in both groups. The lack of statistical significance could reflect the small sample sizes and the heterogeneity of the patients and within each group. Analyses of HIV DNA forms accumulated in non-controllers and controllers provided further support for this observation. Indeed a significant difference was only observed for uDNA, showing that this parameter is better able to highlight differences among the patient groups than total and 2-LTR DNA.

Since total HIV DNA is a rather stable parameter even during ART [63], while 2-LTR circles are more dynamic indicators [64], we further investigated other reservoir-associated dynamic parameters.

Analyses of the ratio of uDNA and 2-LTR circles to total HIV DNA and of 2-LTR circles to uDNA showed differences among the cohorts and heterogeneity among patients within the same cohort. In particular, in non-controllers, uDNA constituted a high portion of total HIV DNA, evidence of active replication in recently infected activated cells [65]. On the contrary, in controllers, there was a lower portion of uDNA forms, which is in line with these patients’ superior and efficient immune and antiviral control. Moreover, the concomitant investigation of uDNA and 2-LTR circle fractions allowed us to speculate on the integrated HIV DNA content, even though this parameter was not directly measured, revealing an overestimation of up to 35% using the 2-LTR assay. Interestingly, we found a high correlation between unintegrated and total HIV DNA in all patient categories, while no association was observed between 2-LTR circles and total HIV DNA. The strong association between uDNA and 2-LTR circles found in HIC patients, in whom the level of uDNA approached that of 2-LTR DNA, suggests that there are presumably no new infection events in HIC and accordingly, we rarely found other unintegrated HIV DNA species apart from 2-LTR circles. These 2-LTR circles may have accumulated in the cells a long time ago and may only contain the correct LTR–LTR junction sequence. On the contrary, in the aviremic On-ART patients, we cannot exclude new rounds of infections leading to the accumulation of other forms of uDNA (both linear and circular) or 2-LTR circles with mutated junction sequences detected only by the U-assay. This could explain the similar levels of 2-LTR circles found in our patient groups using the 2-LTR assay, which presumably detects only a small stable portion of circular DNA with correct junction. Thus, the measurement of 2-LTR circles alone, widely used to evaluate reservoir dynamics, should indeed be used cautiously.

To date, HIC patients have been considered to have large excess amounts of 2-LTR circles and uDNA with low integrated HIV DNA compared to aviremic treated patients [47]. However, the data presented herein show that HIC patients have low uDNA and that 2-LTR circles are a poor correlate of changes in reservoir dynamics. HICs are a heterogeneous group of patients who spontaneously maintain HIV viremia at low or even undetectable levels through different mechanisms, including both viral and host-mediated factors. Our HIC samples were derived from a study in which the EC and LTNP pooled patients were characterized as having a specific subset of NK cells with a specific functional and transcriptional signature that contributed to the containment of the HIV reservoir in terms of total DNA, integrated and unintegrated forms [55].

This study has some limitations. Firstly, several samples had undetectable unintegrated HIV DNA levels. Despite this apparent limitation, qPCR assays have a low quantification limit (2 copies), and therefore. these results do indeed confidently reflect true negative samples when using high input cellular DNA. Second, we quantified total, unintegrated and 2-LTR HIV DNA in whole blood and not in PBMC, as is frequently the case [66–68]. Nevertheless, a high correlation of HIV DNA levels expressed either as copies/10^6^ PBMC, copies/10^6^ CD4+ or copies/mL of whole blood was found by our team (manuscript in preparation) and has been reported by other authors [69]. Moreover, we only assessed HIV DNA in peripheral blood cells, as blood is the most accessible source of cells containing latent HIV-1 and is the most thoroughly studied and understood reservoir, but many other cell types and tissues also contribute to the persistence of HIV DNA and should therefore also be explored [1]. However, carefully selecting the most suitable DNA extraction method allowed us to adapt the U-assay to alternative sample types such as purified CD4+ T cells, PBMC, macrophages [55, 56, 70] and tissue biopsies (Marchetti G. Gut damage and dysfunction: any insight toward an HIV cure? Personal communications. ICAR Italian Conference on AIDS and Retroviruses; 2019). The greater sensitivity of the U-assay compared to the 2-LTR assay demonstrated that in ART-naïve patients, and in patients who were not fully suppressed, other unintegrated forms may have accumulated due to active cycles of replication and that 2-LTR circles constituted less than one-fifth of the unintegrated forms. Moreover, the relative abundance of the various viral DNA forms is dynamic and dependent on the viral conditions of the infection and treatment; hence, quantifying all unintegrated forms, rather than just 2-LTR circles, is the correct approach. Regarding treated patients, it is known that today the majority of these subjects achieve suppression of HIV-1 RNA below 50 copies/mL of plasma [71], and therefore this indicator is not particularly informative as a biomarker of active replication since all patients show roughly the same levels. However, ultrasensitive methods have shown that residual HIV-1 RNA remains detectable in the plasma and stable around a median of 3-10 copies/mL in about 80% of effectively treated patients [72, 73]. Indeed, our results show that the U-assay quantifies all forms of uDNA in the vast majority (76%) of aviremic patients and that this parameter is still informative as it measures any copy number variation, even in the very low range, while HIV-1 RNA remains steadily < 50 copies/mL. A growing body of evidence shows that residual viremia derives from a stable viral reservoir, clonally expanded infected T cells that produce virions, or potentially, from ongoing rounds of infections, or from a combination of all of these factors [74–76]. Thus the presence of 2-LTR circles and/or unintegrated DNA only provides evidence of recent infection events and not necessarily of ongoing virus replication [77]. In any case, it is clear that some degree of replenishment of infected cells exists despite viral suppression below the 50 copies/mL of HIV-1 RNA. Although our U-assay cannot distinguish between uDNA persisting over a long period from uDNA from recent infection, we found an accumulation of unintegrated DNA in infected blood cells during apparently suppressive ART. Furthermore, we showed that this excess is more accurately measured by the U-assay than the 2-LTR assay because in this situation, 2-LTR circles are more likely to be under the quantification limit.

## Conclusion

These findings and the high correlation found between unintegrated and total HIV DNA suggest that the U-assay could be used as a supporting tool to accurately evaluate the DNA reservoir size in blood and its dynamics since uDNA seems to be unaffected or only partially affected by defective genomes, unlike total HIV DNA. The measurement of unintegrated HIV DNA in association with other biomarkers for intracellular HIV DNA and RNA quantification and with conventional plasma HIV-1 RNA levels and CD4+ cell count may lead to improvements in diagnostic methods for ART-patient monitoring and provide support for clinical strategies such as vaccination/eradication and simplification protocols. The potential clinical utility of the quantification of uDNA rather than 2-LTR circles deserves further investigations using a larger cohort of subjects.

## Supplementary information

**Additional file 1: Table S1.** Clinical characteristics of elite controllers (EC) and long-term non-progressors (LTNP) pooled and analyzed as HIV-infected controllers (HIC).

**Additional file 2: Table S2.** Patient sample information.

**Additional file 3: Figure S1.** Sequence analyses of the 2-LTR PCR product in blood DNA samples of HIV-1 infected aviremic On-ART patients. Circle junction sequence lacking the GT dinucleotide (**a**) and anomalous (**b-c**) junction sequences are shown. Three representative sequencing chromatograms are reported.

**Additional file 4: Table S3.** Correlations between the levels of integrated HIV DNA (iDNA) and uDNA in blood samples fron HIV-1 infected patients. **Table S4.** Correlations between plasma HIV-1 RNA and the levels of the different forms of HIV DNA in blood samples from HIV-1 infected patients. **Table S5.** Correlations between CD4+ T cell count and the levels of the different forms of HIV DNA in blood samples from HIV-1 infected patients.

## Data Availability

All data generated or analyzed during this study are included in this published article and its additional files.
