# Supplementary material for: A comparative analysis of unintegrated HIV-1 DNA measurement as a potential biomarker of the cellular reservoir in the blood of patients controlling and non-controlling viral replication
Source: J Transl Med. 2020 May 19;18:204. doi: 10.1186/s12967-020-02368-y (PMC7236182; doi:10.1186/s12967-020-02368-y)
Supplement: Supplementary file 1 — Additional file 1: Table S1. Clinical characteristics of elite controllers (EC) and long-term non-progressors (LTNP) pooled and analyzed as HIV-infected controllers (HIC). [file 12967_2020_2368_MOESM1_ESM.docx]

**Table S1.** Clinical characteristics of elite controllers (EC) and long-term non-progressors (LTNP) pooled and analyzed as HIV-infected controllers (HIC)

|  | EC  (n=8) | LTNP  (n=12) | Mann Whitney test  (P value) |
| --- | --- | --- | --- |
| CD4+ T count (cells/µl) |  |  |  |
| Median | 913 | 736 | 0.512 |
| IQR | 578-1231 | 607-1014 |  |
| HIV-1 RNA (cp/ml plasma) |  |  |  |
| Median | 10 | 549 | 0.0011 |
| IQR | 0-39.5 | 305-1903 |  |
| Total HIV DNA |  |  |  |
| Median (cp/µg DNA) | 7.5 | 7.5 | 0.9077 |
| IQR | 3.5-12.5 | 2.3-12.5 |  |
| Median (cp/10^4^ CD4+) | 5.4 | 5.4 | 0.9385 |
| IQR | 2.6-7.0 | 1.9-9.0 |  |
| uDNA |  |  |  |
| Median (cp/µg DNA) | 1.0 | 1.5 | 0.6065 |
| IQR | 1.0-2.0 | 1.0-2.0 |  |
| Median (cp/10^4^ CD4+) | 1.0 | 1.0 | 0.5771 |
| IQR | 1.0-1.8 | 1.0-1.0 |  |
| 2-LTR circles |  |  |  |
| Median (cp/µg DNA) | 1.0 | 1.0 | 0.9294 |
| IQR | 1.0-2.0 | 1.0-2.0 |  |
| Median (cp/10^4^ CD4+) | 1.0 | 1.0 | 0.4972 |
| IQR | 1.0-1.7 | 1.0-1.2 |  |
| % of uDNA vs Total HIV DNA |  |  |  |
| Median | 0 | 8 | 0.5922 |
| IQR | 0-25 | 0-23 |  |
| % of 2-LTR vs Total HIV DNA |  |  |  |
| Median | 0 | 0 | 0.9633 |
| IQR | 0-22.75 | 0-18 |  |
| % 2-LTR vs uDNA |  |  |  |
| Median | 89 | 77 | 0.2619 |
| IQR | 84-100 | 48-92 |  |
