# Supplementary figures and images for: A comparative analysis of unintegrated HIV-1 DNA measurement as a potential biomarker of the cellular reservoir in the blood of patients controlling and non-controlling viral replication
Source: J Transl Med. 2020 May 19;18:204. doi: 10.1186/s12967-020-02368-y (PMC7236182; doi:10.1186/s12967-020-02368-y)

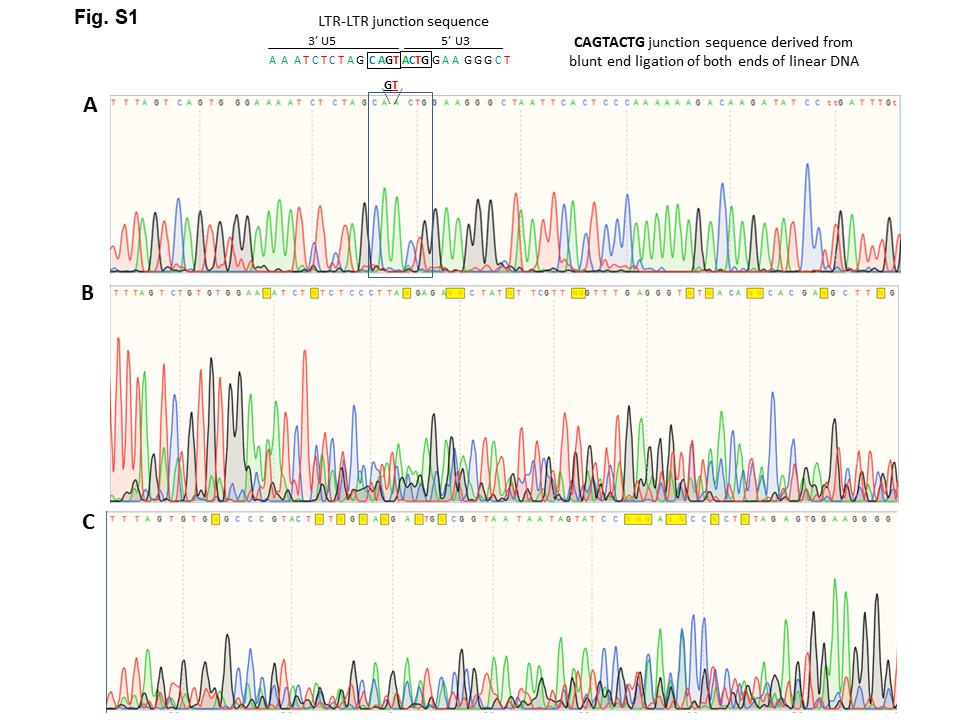

Supplement: Supplementary file 3 — Additional file 3: Figure S1. Sequence analyses of the 2-LTR PCR product in blood DNA samples of HIV-1 infected aviremic On-ART patients. Circle junction sequence lacking the GT dinucleotide (a) and anomalous (b-c) junction sequences are shown. Three representative sequencing chromatograms are reported. [file 12967_2020_2368_MOESM3_ESM.tif]
