# Supplementary material for: A comparative analysis of unintegrated HIV-1 DNA measurement as a potential biomarker of the cellular reservoir in the blood of patients controlling and non-controlling viral replication
Source: J Transl Med. 2020 May 19;18:204. doi: 10.1186/s12967-020-02368-y (PMC7236182; doi:10.1186/s12967-020-02368-y)
Supplement: Supplementary file 4 — Additional file 4: Table S3. Correlations between the levels of integrated HIV DNA (iDNA) and uDNA in blood samples fron HIV-1 infected patients. Table S4. Correlations between plasma HIV-1 RNA and the levels of the different forms of HIV DNA in blood samples from HIV-1 infected patients. Table S5. Correlations between CD4+ T cell count and the levels of the different forms of HIV DNA in blood samples from HIV-1 infected patients. [file 12967_2020_2368_MOESM4_ESM.doc]

**Additional file 4**

**Table S3.** Correlations between the levels of integrated HIV DNA (iDNA) and uDNA in blood samples from HIV-1 infected patients

|  | Correlation coefficient | P values |
| --- | --- | --- |
| Overall | 0.48 | **0.000** |
| Off-ART | 0.60 | **0.038** |
| Viremic On-ART | 0.26 | 0.362 |
| Aviremic On-ART | 0.33 | 0.076 |
| HIC | 0.34 | 0.142 |

**Table S4.** Correlations between plasma HIV-1 RNA and the levels of the different forms of HIV DNA in blood samples from HIV-1 infected patients

|  | Total HIV DNA | |  | uDNA | |  | 2-LTR circles | |
| --- | --- | --- | --- | --- | --- | --- | --- | --- |
|  | Correlation coefficient | P values |  | Correlation coefficient | P values |  | Correlation coefficient | P values |
| Overall | 0.10 | 0.379 |  | 0.20 | 0.086 |  | 0.04 | 0.722 |
| Off-ART | 0.65 | **0.023** |  | 0.75 | **0.005** |  | 0.34 | 0.283 |
| Viremic On-ART | 0.24 | 0.400 |  | -0.02 | 0.949 |  | -0.36 | 0.200 |
| Aviremic On-ART | -0.37 | 0.055 |  | -0.25 | 0.182 |  | 0.35 | 0.063 |
| HIC | 0.38 | 0.101 |  | 0.22 | 0.353 |  | 0.17 | 0.468 |

**Table S5.** Correlations between CD4+ T cell count and the levels of the different forms of HIV DNA in blood samples from HIV-1 infected patients

|  | Total HIV DNA | |  | uDNA | |  | 2-LTR circles | |
| --- | --- | --- | --- | --- | --- | --- | --- | --- |
|  | Correlation coefficient | P values |  | Correlation coefficient | P values |  | Correlation coefficient | P values |
| Overall | -0.68 | **0.000** |  | -0.59 | **0.000** |  | -0.28 | **0.016** |
| Off-ART | -0.63 | **0.028** |  | -0.37 | 0.236 |  | -0.13 | 0.685 |
| Viremic On-ART | -0.51 | **0.050** |  | -0.50 | 0.070 |  | -0.22 | 0.441 |
| Aviremic On-ART | -0.68 | **0.000** |  | -0.23 | 0.221 |  | -0.35 | 0.064 |
| HIC | -0.46 | **0.043** |  | 0.06 | 0.804 |  | 0.09 | 0.711 |
